# Charge transfer complexation boosts molecular conductance through Fermi level pinning†
†Electronic supplementary information (ESI) available: Synthetic procedures and characterisation transcripts for the compounds used in this study. Additional single-molecule conductance data and further analysis. Details on the theoretical calculations. See DOI: 10.1039/c8sc04199g


**DOI:** 10.1039/c8sc04199g

**Published:** 2019-01-03

**Authors:** Kun Wang, Andrea Vezzoli, Iain M. Grace, Maeve McLaughlin, Richard J. Nichols, Bingqian Xu, Colin J. Lambert, Simon J. Higgins

**Affiliations:** a Department of Physics and Astronomy & NanoSEC , University of Georgia , 220 Riverbend Road , Athens , GA 30602 , USA . Email: bxu@engr.uga.edu; b Department of Chemistry , University of Liverpool , Crown Street , Liverpool L69 7ZD , UK . Email: shiggins@liverpool.ac.uk; c Department of Physics , Lancaster University , Lancaster LA1 4YB , UK . Email: c.lambert@lancaster.ac.uk; d College of Engineering & NanoSEC , University of Georgia , 220 Riverbend Road , Athens , GA 30602 , USA

## Abstract

Interference features in the transmission spectra can dominate charge transport in metal–molecule–metal junctions when they occur close to the contact Fermi energy (*E*_F_). Here, we show that by forming a charge-transfer complex with tetracyanoethylene (TCNE) we can introduce new constructive interference features in the transmission profile of electron-rich, thiophene-based molecular wires that almost coincide with *E*_F_. Complexation can result in a large enhancement of junction conductance, with very efficient charge transport even at relatively large molecular lengths. For instance, we report a conductance of 10^–3^*G*_0_ (∼78 nS) for the ∼2 nm long α-quaterthiophene:TCNE complex, almost two orders of magnitude higher than the conductance of the bare molecular wire. As the conductance of the complexes is remarkably independent of features such as the molecular backbone and the nature of the contacts to the electrodes, our results strongly suggest that the interference features are consistently pinned near to the Fermi energy of the metallic leads. Theoretical studies indicate that the semi-occupied nature of the charge-transfer orbital is not only important in giving rise to the latter effect, but also could result in spin-dependent transport for the charge-transfer complexes. These results therefore present a simple yet effective way to increase charge transport efficiency in long and poorly conductive molecular wires, with important repercussions in single-entity thermoelectronics and spintronics.

## Abstract 2

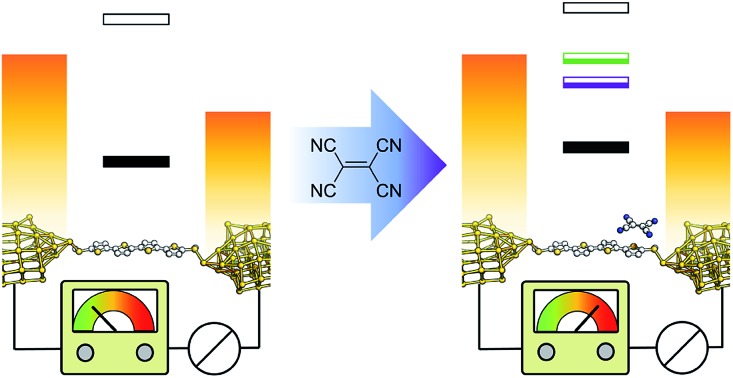
Efficient charge transport across long molecular wires enabled by charge-transfer complexation, through Fermi level pinning of interference features.

## Introduction

The ability to manipulate quantum transport through single molecules is key to building functional molecular electronic devices. Towards this goal, various approaches have been explored in metal–molecule–metal junctions to exert control on molecular conductance, including optical illumination,^1^–^3^ gating the electrochemical^4^–^7^ or electrostatic^8^–^11^ environment, and through reversible supramolecular interactions such as solvent effects,^12^–^16^ complexation,^17^–^24^ π-stacking interactions^25^–^31^ and hydrogen-bonding.^32^–^34^ Another way that has been proposed to chemically control the charge-transport properties of a molecular junction is by harnessing quantum interference phenomena with energy close to the electrode Fermi level *E*_F_. Drawing inspiration from the seminal work of Sautet and Joachim,^35^ destructive interferences have been introduced, for instance, by exploiting meta-connectivity in π-conjugated systems,^36^–^39^ cross-conjugation,^40^–^42^ or the unique σ-bonding properties of organosilanes,^43^ and these have been employed to lower molecular conductance for the fabrication of efficient molecular insulators. In addition to providing a way to control molecular conductance, the presence of such transport features also results in higher Seebeck coefficients (a measure of thermoelectric conversion) at room temperature,^44^ which is a highly sought-after and promising property of molecular nanodevices. The conductance decrease associated with destructive interferences, however, reduces the power factor and figure of merit of these devices, thereby limiting their suitability for functional devices. Constructive interference in quantum transport have been well known in the physics literature, since the classic paper of Breit and Wigner,^45^ and transmission resonances in 1-dimensional systems have been widely studied both in the presence of many scatterers^46^–^48^ and in the case of a single scatterer.^35^ Conductance in multi-path structures was examined from a physical^49^,^50^ and chemical perspective.^51^ The latter suggested that when two conducting paths are connected in parallel within a molecule, the resulting conductance should be increased by a factor of four, rather than the factor of two observed in classical electrical networks. The prediction was later experimentally tested^52^,^53^ by measuring the conductance of analogous pairs of double-backbone and single-backbone molecular junctions, and their conductance ratio was found to lie between 3.3 and 1.6 (*i.e.* ratios were either higher or lower than the classical value of two, but did not reach the predicted ratio of four). Reliable experimental strategies for the introduction of constructive quantum interference features resulting in large conductance boosts are therefore lacking or inefficient. As a consequence, the development of robust methods to substantially increase conductance through *constructive* interference would provide valuable strategies for the design of molecular systems for efficient charge transport and thermoelectric conversion.

Among the supramolecular interactions mentioned earlier, charge-transfer (CT) complexation is of particular interest from the point of view of electronic devices. Several ground-breaking discoveries, such as the first organic material with metallic behaviour (TTF:TCNQ)^54^ and the first organic superconductor (Bechgaard salt, [TMTSF]_2_PF_6_)^55^ used CT complexes as bulk conductors, and complexation in nanoscale junctions has been used to gate molecular conductance.^17^ We further explored the application of CT complexes in single-molecule electronics and we report here a general approach to the introduction of *constructive* quantum interference that can be applied to conventional, fully conjugated, molecular wires. CT complexation introduces additional resonances in their transmission profile, and these resonances have a Fano line-shape (as opposed to the more common Breit–Wigner distribution) characterised by an antiresonance followed by a sharp positive contribution. As these features arise from a part-filled orbital, their energetic position is predicted by simple band theory to be always near to the Fermi level of the electrodes *E*_F_. The presence of a transport feature near *E*_F_ in the transmission profile is of the utmost importance for the development of both long-range tunnelling and highly efficient thermoelectric power conversion at room temperature, and the ability to reliably pin it at the desired energy level is also a very sought-after property in molecular wire design. We demonstrate empirically that the Fano resonance introduced by the formation of a charge-transfer complex of a molecular wire with ethenetetracarbonitrile (TCNE or “tetracyanoethylene”) is reliably placed at energy values close to the Fermi level of the electrodes (“pinning”), which results in complexes having conductance almost independent of the nature of the molecular backbone and contacts to the electrodes. The pinning effect results in a greatly reduced attenuation coefficient *β* of the complexes across an oligomeric thienyl series, yielding efficient charge transport and large conductance values even through relatively long molecular wires.

## Results and discussion

**Fig. 1:**
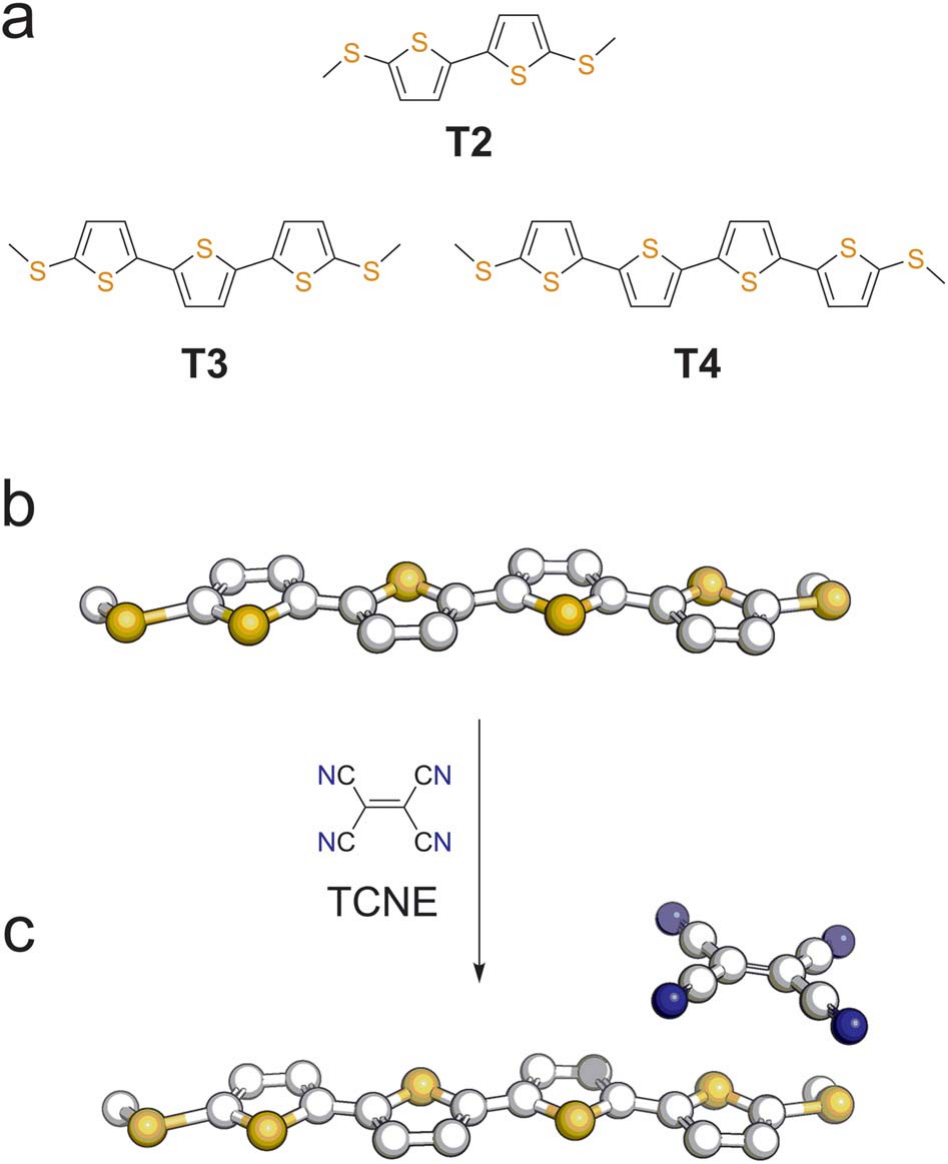
(a) Structures and naming of the compounds used in this study. Representation of a molecular wire (b) and its TCNE complex (c).

We focussed our study on a series of oligothiophenes, terminated at each end with methyl thioether contacts. Oligothiophenes were chosen as electron-rich heteroaryl units, which are known to form 1 : 1 CT complexes with strong electron acceptors such as TCNE.^56^–^58^ The compounds used in this study are presented in Fig. 1a, and details of the synthetic procedures can be found in the ESI.†

**Fig. 2:**
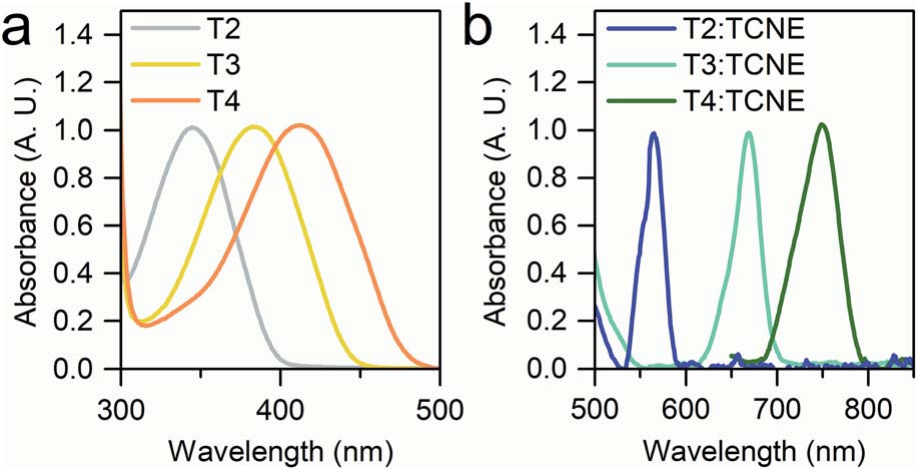
UV-vis spectra of the **T2–T4** series (a) and of their TCNE complexes (b) in CH_2_Cl_2_. A 1 : 100 ratio of molecule : TCNE was used to obtain (b). All the spectra are normalised, and the onset of the **T4** π → π* band below 650 nm in (b) was removed for clarity. The CT bands are two orders of magnitude less intense than the π → π* transition.

We started our investigation by assessing the physical properties of the charge transfer complexes of these oligothiophene-based molecular wires, in order to determine their suitability for single-molecule conductance studies. As shown in Fig. 2, UV-vis spectroscopy in CH_2_Cl_2_ shows that an absorption band in the 550–800 nm region, corresponding to the transition, arises upon complexation, confirming the formation of the charge-transfer complex.

**Fig. 3:**
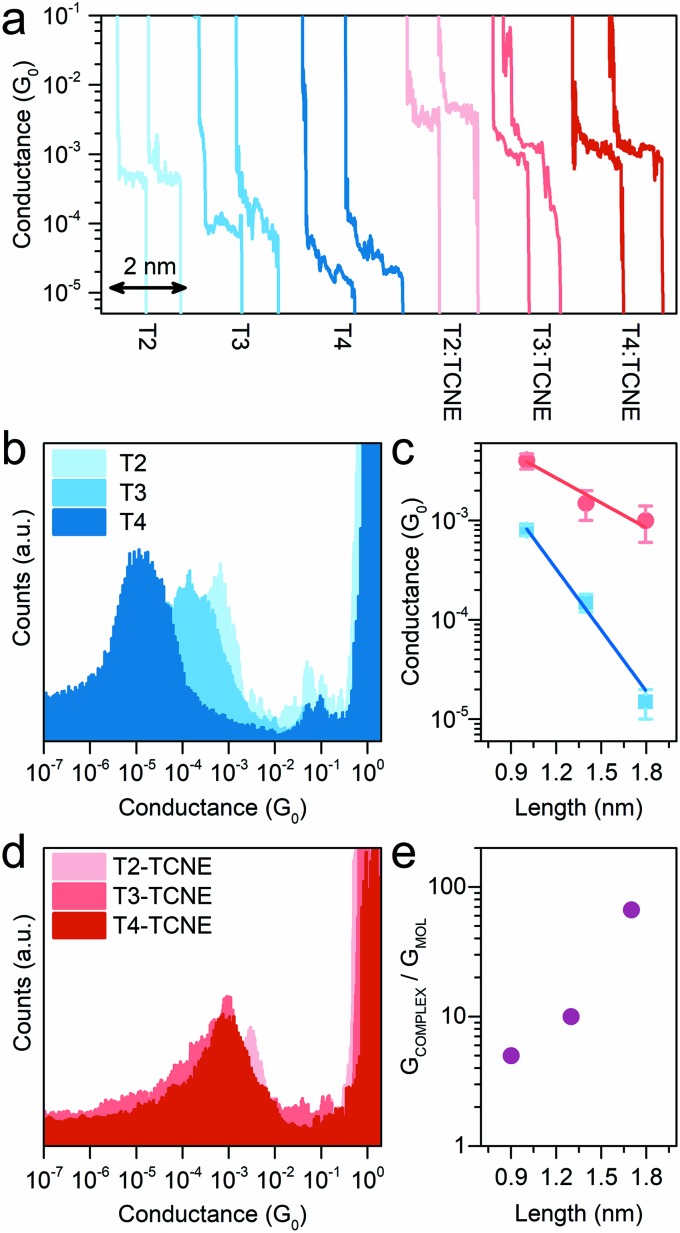
Length-dependent conductance measurements of methyl-thioether terminated oligothiophenes with 2 (**T2**), 3 (**T3**) and 4 (**T4**) thiophene units. Example conductance *vs.* electrode displacement traces (a) of the isolated molecules (blue shades – from left to right: **T2**, **T3** and **T4**) and the corresponding complexes with TCNE (red shades – from left to right: **T2**:TCNE, **T3**:TCNE and **T4**:TCNE). Conductance histograms of **T2**, **T3** and **T4** (b) and their TCNE complexes (d). Conductance *vs.* molecular length plot (c) for the isolated molecules (blue) and the respective complexes with TCNE (red) and conductance enhancement *vs.* molecular length plot (e). Error bars in (c) represent the standard deviation. Molecular length calculated at DFT level (see Methods).

Having established the fact that these thiomethyl-terminated oligothiophenes form CT complexes with TCNE, the scanning tunnelling microscopy-break junction (STM-BJ) technique^59^ was then used to fabricate and measure the electrical properties of molecular junctions. In brief, a Au STM tip was brought into contact with a Au(111) substrate, in the presence of either the sample molecule or its TCNE complex (prepared as described in the Methods section), as a pre-adsorbed sub-monolayer. The tip was then retracted while the tunnelling current was monitored. As the tip is crashed into the substrate, a fresh junction is formed, and on retraction this thins down to a single atom (point contact, characterised by its conductance *G*_0_ ≈ 77.48 μS), which is finally broken upon further withdrawal. When the point contact breaks, if a molecule (or molecules) binds to both Au contacts, then subsequent additional plateaux are seen in the current–distance plot at values much smaller than *G*_0_, corresponding to charge transport through the molecular bridge. Eventually, as retraction continues, the molecular junction breaks and the current abruptly falls to a very low value consistent with direct electrode tunnelling. Thousands of such traces are recorded for each set of experimental conditions, and the traces are compiled into conductance histograms. The plateaux result in peaks in the histogram at values below *G*_0_, which represent the distribution of conductance values of the molecular junction, and the same traces are used for further plateau length analysis. More details can be found in the Methods section and in the ESI,† and the conductance measurements are presented in Fig. 3.

The non-complexed oligothiophene series showed, as expected, an exponential decay of the conductance with increasing molecular length, with an associated decay constant *β* = 5.0 nm^–1^, in reasonable agreement with earlier data reported on the same system (4 nm^–1^).^60^ The small differences in the absolute conductance values and in *β* can be attributed to the different media used for the STM-BJ measurements, which is air in this study and 1,2,4-trichlorobenzene in Capozzi *et al.* It has already been demonstrated that the junction behaviour can be greatly influenced by the solvent used during the measurements.^12^,^14^–^16^,^61^ The conductance decay upon exposure to TCNE, on the other hand, showed a greatly reduced attenuation factor *β* of 1.7 nm^–1^, highlighting the almost length-independent conductance of the charge-transfer complexes, which lies relatively constant at approximately 10^–3^*G*_0_. This allows efficient charge transport even at large molecular lengths (10^–3^*G*_0_ conductance through a ∼2 nm molecular wire). Furthermore, the conductance boost upon complexation (as *G*_COMPLEX_/*G*_MOL_) follows an exponential increase as predicted. A summary of all conductance values can be found in Table 1.

**Table 1:** Summary of single-molecule conductance values of the compounds presented in this study and their charge-transfer complexes with TCNE. Theoretical values are presented in brackets

Compound	Molecular conductance (*G*_0_)	TCNE complex conductance (*G*_0_)	Ratio: *G*_COMPLEX_/*G*_MOL_
**T2**	8 × 10^–4^ (3 × 10^–3^)	4 × 10^–3^ (8 × 10^–3^)	∼5(2)
**T3**	1.5 × 10^–4^ (4 × 10^–4^)	1.5 × 10^–3^ (2 × 10^–3^)	∼10(5)
**T4**	1.5 × 10^–5^ (3 × 10^–5^)	1 × 10^–3^ (1 × 10^–3^)	∼67(33)

To rule out the possibility that the source of the high conductance feature consistently near ∼10^–3^*G*_0_ is from TCNE itself, conductance measurements on samples either with no molecule or solely with TCNE were therefore performed as a control. No significant feature was found in the conductance histograms within the range of 10^–5^ to 1 *G*_0_ (see ESI, Fig. S3†). In addition, plateau length analysis revealed that the molecule:TCNE junctions have break-off distances similar to those of the corresponding uncomplexed molecular wires (see ESI, Fig. S2†), further excluding the contribution of TCNE alone. We thus conclude that the conductance peak at ∼10^–3^*G*_0_ observed from all molecule:TCNE samples is mainly contributed by the formation of molecule:TCNE complexes due to CT complexation.

To understand the observed phenomena, the junction behaviour was then modelled with DFT, focussing on a dynamics study of the geometrical fluctuations of the molecular wire trapped in the junction and of its TCNE complex. The zero-bias transmission coefficient *T*(E) was obtained for 500 different configurations, sampling a range of structures of the CT complex in the junction, and the average finite-bias room-temperature conductance was then calculated (details in the Methods section and examples of structures and relative density of states plots in the ESI†). This approach was previously used^17^ to capture a realistic picture of the complexed molecular junction at room temperature, accounting for the fluctuations in the TCNE orientation and position with respect to the oligothiophene backbone. Spectroscopic and crystallographic data on CT complexes of oligothiophenes consistently report a 1 : 1 complex ratio,^57^,^62^,^63^ and this was used in our calculations. An additional Fano resonance^64^ has been previously observed in the DFT-calculated transmission curves for the TCNE complex of a bis(thialkyl)terthiophene^17^ which accounted for the conductance boost phenomenon, and similar resonances arise with TCNE complexation of the oligomeric series **T2–T4**. In the CT complexes, the donation of partial electron density to TCNE results in part-filled orbitals on the molecular backbone and TCNE itself, the latter acting as a scattering centre and generating the Fano resonance through quantum interference and accounting for the observed conductance change. The asymmetric line shape of Fano resonances^36^,^64^ occurs when a backbone orbital coupled to a continuum of states in the electrodes interacts with a pendant orbital, which in our case resides on the TCNE.

**Fig. 4:**
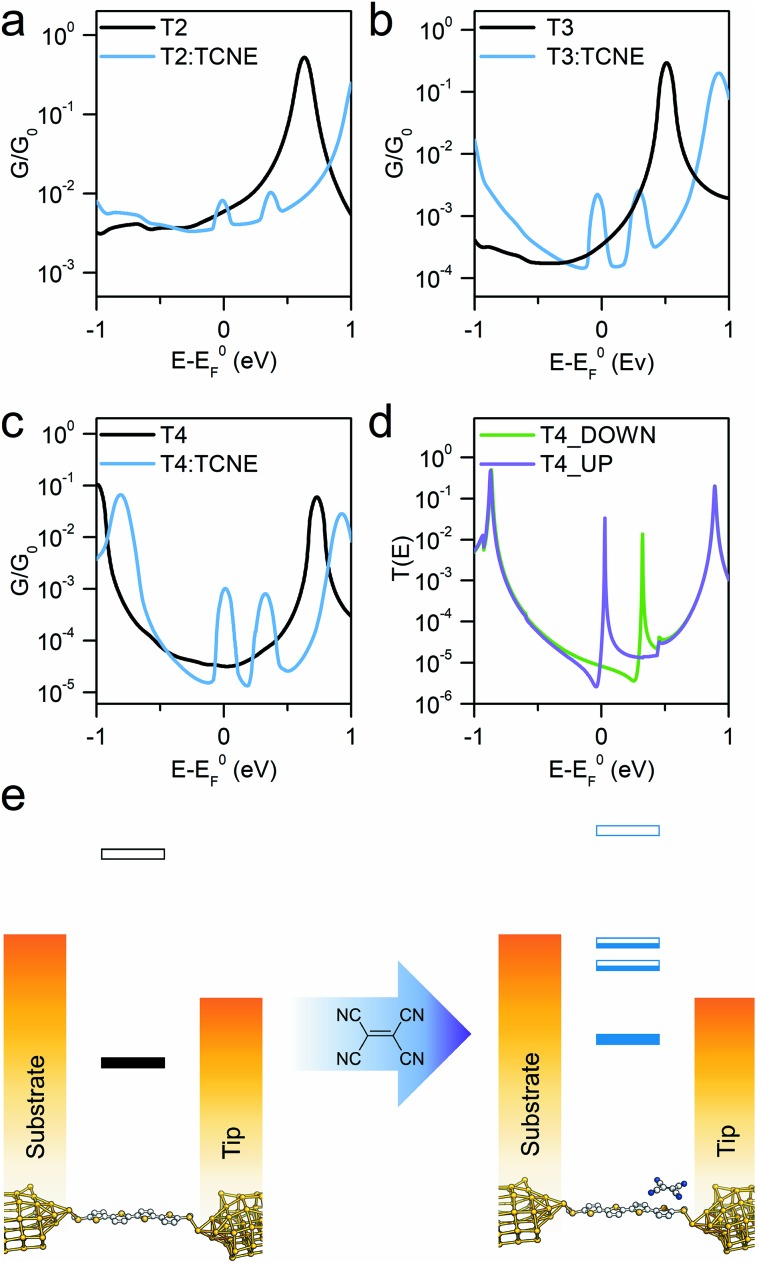
Room temperature calculated conductance for the isolated molecules (black line) and complexed with TCNE (blue) for compounds **T2** (a), **T3** (b) and **T4** (c), along with a representative spin-polarised *T*(E) curve for the TCNE complex of **T4** (d). Schematic diagram (e) showing the position of the DFT resonances for **T4** respective to the two electrodes before and after TCNE complexation. The part-filled CT resonances in the HOMO–LUMO gap are responsible for the observed increase in conductance.

The sharp resonances in the individual transmission curves result in “bumps” in the thermally-averaged finite-bias calculated conductance, very near to *E*_F_ in energy (Fig. 4), which are responsible for the observed increase in conductance upon complexation. Calculations predict a *β* value for the “naked” molecular wires of 6.5 nm^–1^ at the DFT predicted Fermi energy, and the complexed series **T2–T4** give a value of 1.2 nm^–1^, both in reasonable agreement with the experimental findings. The theoretically predicted conductance enhancement ratios upon TCNE complexation are slightly lower than the experimental value due to the calculated values for the isolated molecule being slightly higher than the measured ones, but the results are consistent with the experimental trend. The key aspect of the interpretation of the experimental results is that DFT predicts the position of these additional interference features always close (“pinned”) to *E*_F_. The reason behind this lies in the strength of TCNE as electron acceptor, which forces the position of the part-filled orbital responsible for the Fano resonance automatically to adjust in order to achieve the required filling. This phenomenon also accounts for the relative independence of the interference feature energy on the location and orientation of the TCNE relative to the molecular backbone, which results in the well-defined “bumps” observed in the calculated conductance *vs.* energy curves, obtained as average of 500 different *T*(E) curves. As the interference feature is reliably placed near *E*_F_, its contribution dominates charge transport and therefore the charge-transfer complex conductance is only a function of the room temperature fluctuations in the energy position of the zero-Kelvin Fano resonance (average room temperature *G*(E) curves in Fig. 4a–c). Furthermore, as can be observed in Fig. 4d, there is significant spin splitting in the transmission curve, and this is again due to the partly-filled nature of the orbital associated with the molecule → TCNE charge transfer, behaving similarly to an unpaired electron. Spin splitting results in the majority-spin contribution moving closer to *E*_F_ and the minority-spin contribution shifting away, and this phenomenon is responsible for the double bump in the calculated conductance curves (Fig. 4a–c). A similar behaviour was found in **T2** and **T3** (ESI, Fig. S5†). Therefore, the conductance at *E*_F_ is predicted to be different for the two spins, which could result in magnetoresistance effects.

As the CT resonance pinned to electrode *E*_F_ dominates charge transport through these molecular wires, one can postulate that such effect would not be affected even when changing the molecule-electrode contacts of the junction. As further evidence for the proposed mechanism of pinning of the CT transport resonance, we fabricated and characterised two additional α-terthienyl compounds bearing thiol contacts. The same conductance measurements were carried out for these molecules. The results again showed conductance pinned at 10^–3^*G*_0_ when complexed with TCNE (see ESI, Fig. S1†) even though their uncomplexed state gave completely different conductance values (ranging from 10^–5^*G*_0_ to 10^–4^*G*_0_). These additional results further support the proposed mechanism, with the conductance of molecule:TCNE complex confirmed independent of the contacts to the electrodes. The overall experimental and computational results confirm that (I) the energetic position of the charge-transfer interference feature is pinned at the Fermi level of the metallic electrodes and (II) this interference pinning phenomenon greatly reduces the attenuation coefficient *β*, thus enabling efficient charge transport even through relatively long molecular wires.

## Conclusions

In summary, we have shown here that charge-transfer complexation of a series of oligothiophene molecular wires with TCNE leads to an up to ∼70-fold increase in molecular junction conductance. The resulting complexes have conductance values nearly independent of the nature of the molecular backbone and contacts to the electrode and show a greatly reduced attenuation coefficient *β* of 1.7 nm^–1^, enabling long-range efficient tunnelling. DFT calculations show that this phenomenon arises from constructive quantum interference features in the transmission profile (Fano resonances) pinned to the electrode Fermi level, which arise upon complexation. The conductance boost of more than one order of magnitude observed in the longer molecular wires, such as α-quaterthiophene, not only offers a straightforward way to greatly enhance electrical conductance, but also makes these compounds and the CT complexation phenomenon interesting for the development of sensors with single-molecule sensitivity. Furthermore, a sharp transmission feature near the electrode Fermi level is a long-anticipated feature for the development of molecular thermoelectric devices with high Seebeck coefficient and figure of merit. Overall, this approach charts a clear path for the fabrication of molecular devices with some of the most desirable features, such as high conductance and long-range transport, with a high predicted efficiency in energy conversion. This study further stresses the importance of interference features in the transmission profile and the unique properties of nanoscale junctions operating in the quantum realm, where even weak supramolecular interactions can have a dramatic effect on junction behaviour.

## Methods

### Synthesis

**T3** was prepared from 2,2′:5′,2′′-terthiophene by treatment with two equivalents of *n*-butyllithium, followed by quench with dimethyl disulfide. **T2** was prepared from 2,2′-bithiophene by treatment with two equivalents of *n*-butyllithium, followed by quench with S_8_/MeI. **T4** was prepared by Stille coupling of 5,5′-dibromo-2,2′-bithiophene and trimethyl(5-(methylthio)thiophen-2-yl)stannane. Synthetic procedures and NMR/HRMS/CNH data are provided in the ESI.†

### Characterisation

UV/vis spectra were recorded in CH_2_Cl_2_ on a double beam PerkinElmer λ25 spectrometer, using a 1 cm quartz cell at room temperature.

### Conductance measurements

The conductance of molecular junctions were determined using the STM break junction (STM-BJ) method.^59^ Au substrates were prepared by evaporating ∼100 nm of gold onto freshly cleaved mica sheets using a thermal evaporator under a vacuum of 10^–7^ Torr. The gold beads for Au substrate deposition was purchased from Kurt J. Lesker Company (99.999%), and mica sheets were purchased from Ted Pella, Inc. Freshly annealed Au surfaces were incubated in solutions of the desired molecular wire (10^–3^ M, CH_2_Cl_2_ : Ethanol 1 : 1) for 30 minutes to allow adsorption of a sub-monolayer of the molecular wires, and then rinsed with ethanol and dried with high purity nitrogen gas. To form the TCNE complexes, a TCNE solution (10^–2^ M in CH_2_Cl_2_) was dropped onto the prepared sub-monolayer of sample molecules and incubated for 1 h. Samples were then copiously rinsed with ethanol and dried with high purity nitrogen gas. Conductance measurements were carried out in air at room temperature (∼20 °C), with a bias of 100 mV applied to the substrate (tip grounded). Conductance data was collected by driving a freshly cut Au tip into a freshly annealed Au substrate (35 nm s^–1^) and then withdrawing it at constant speed (20 nm s^–1^). As the tip was pushed into the surface and then retracted, a fresh Au–Au junction was formed, thinned down to a single atom (point contact), and finally broken upon further retraction. After the rupture of the junction, a molecule can bridge the tip-substrate gap. The tip was engaged towards the Au substrate until the tip-substrate current was 25 μA, and then crashed into the substrate a further 0.5 nm to ensure good physical contact between the tip and substrate before being withdrawn. This setting guaranteed that a clear *G*_0_ point contact plateau is present in each trace. The current (*I*) was recorded at a fixed tip-substrate bias (*V*) and conductance *G* is determined by Ohm's law (*G* = *I*/*V*). The process was repeated thousands of times and the resulting current *vs.* distance traces were used for the construction of conductance histograms.

### Theoretical conductance calculations

The optimised geometry of each molecule and TCNE was calculated using the density functional code SIESTA.^65^ A double-ζ basis set was used along with norm-conserving pseudopotentials. The energy cut-off to determine the fineness of the real space grid was 150 Rydbergs and the exchange correlation was described by GGA.^66^ The molecules were then relaxed until all forces were less than 0.01 eV Å^–1^. The binding of the molecules to gold electrodes was then calculated by a further geometry optimisation. A gold electrode consisting of a pyramid of 4 gold atoms attached to 8 layers of Au(111) each containing 9 atoms was constructed and the molecule attached through the anchor group, in the case of the thioacetate-protected molecules the acetyl group attached to the sulfur was removed. The resulting binding geometries show the binding distances between the terminal Au atom and the S atom of the anchor group to be 2.5 Å (**T2–T4**) and the Au–S–C angles to be 120° (**T2–T4**). To model the addition of TCNE molecules to the junction we calculate the binding energy due to the formation of a charge transfer complex in each of the three molecules. Firstly, the optimum binding location is found by moving the TCNE across the backbone and locating the binding energy minimum. This geometry was then used to perform a geometry relaxation to give the optimised geometry of the complex. The shape and position of the Fano resonance is dependent on the binding geometry between the TCNE and molecule so, to account for geometry fluctuations following the previous theoretical model,^17^ 500 geometries of the TCNE molecule are taken in the vicinity of the optimum position. The junction geometries are then utilised in the DFT code SIESTA to generate a Hamiltonian. The extended molecule in each case is constructed with the gold electrodes incorporating 8 layers of Au(111) with each layer containing 9 atoms, and the quantum transport code GOLLUM^67^ was then used to calculate the spin-polarised zero-bias transmission coefficient which is related to the electrical conductance through the Landauer formalism. We then calculate the conductance at room temperature by evaluating:
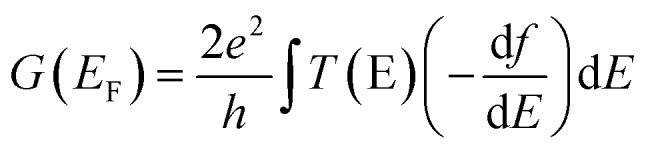
where *f* is the Fermi distribution. For the complexed molecules an average is taken over all calculated geometries.

## Data availability

Proton and ^13^C NMR spectra in Bruker data format for the compounds used in this study are deposited in the University of Liverpool data catalogue (collection #464) at the address ; http://datacat.liverpool.ac.uk/464/ and at DOI: ; 10.17638/datacat.liverpool.ac.uk/464.

Single-molecule conductance and DFT data is available from the authors upon request.

## Conflicts of interest

There are no conflicts to declare.

## Supplementary Material

Supplementary information

## References

[cit1] Fung E. D., Adak O., Lovat G., Scarabelli D., Venkataraman L. (2017). Nano Lett..

[cit2] Yoshida K., Shibata K., Hirakawa K. (2015). Phys. Rev. Lett..

[cit3] Zhou J., Wang K., Xu B., Dubi Y. (2018). J. Am. Chem. Soc..

[cit4] Osorio H. M., Catarelli S., Cea P., Gluyas J. B. G., Hartl F., Higgins S. J., Leary E., Low P. J., Martín S., Nichols R. J., Tory J., Ulstrup J., Vezzoli A., Milan D. C., Zeng Q. (2015). J. Am. Chem. Soc..

[cit5] Li Z., Pobelov I., Han B., Wandlowski T., Błaszczyk A., Mayor M. (2007). Nanotechnology.

[cit6] Baghernejad M., Manrique D. Z., Li C., Pope T., Zhumaev U., Pobelov I., Moreno-García P., Kaliginedi V., Huang C., Hong W., Lambert C., Wandlowski T. (2014). Chem. Commun..

[cit7] Brooke R. J., Jin C., Szumski D. S., Nichols R. J., Mao B., Thygesen K. S., Schwarzacher W. (2015). Nano Lett..

[cit8] Kim Y., Jeong W., Kim K., Lee W., Reddy P. (2014). Nat. Nanotechnol..

[cit9] Xiang D., Jeong H., Kim D., Lee T., Cheng Y., Wang Q., Mayer D. (2013). Nano Lett..

[cit10] Park J., Pasupathy A. N., Goldsmith J. I., Chang C., Yaish Y., Petta J. R., Rinkoski M., Sethna J. P., Abruña H. D., McEuen P. L., Ralph D. C. (2002). Nature.

[cit11] Aragonès A. C., Haworth N. L., Darwish N., Ciampi S., Bloomfield N. J., Wallace G. G., Diez-Perez I., Coote M. L. (2016). Nature.

[cit12] Fatemi V., Kamenetska M., Neaton J. B., Venkataraman L. (2011). Nano Lett..

[cit13] Leary E., Höbenreich H., Higgins S. J., van Zalinge H., Haiss W., Nichols R. J., Finch C., Grace I., Lambert C. J., McGrath R., Smerdon J. (2009). Phys. Rev. Lett..

[cit14] Choi B., Capozzi B., Ahn S., Turkiewicz A., Lovat G., Nuckolls C., Steigerwald M. L., Venkataraman L., Roy X. (2016). Chem. Sci..

[cit15] Milan D. C., Al-Owaedi O. A., Oerthel M.-C., Marqués-González S., Brooke R. J., Bryce M. R., Cea P., Ferrer J., Higgins S. J., Lambert C. J., Low P. J., Manrique D. Z., Martin S., Nichols R. J., Schwarzacher W., García-Suárez V. M. (2016). J. Phys. Chem. C.

[cit16] Nakashima S., Takahashi Y., Kiguchi M. (2011). Beilstein J. Nanotechnol..

[cit17] Vezzoli A., Grace I., Brooke C., Wang K., Lambert C. J., Xu B., Nichols R. J., Higgins S. J. (2015). Nanoscale.

[cit18] García R., Herranz M. Á., Leary E., González M. T., Bollinger G. R., Bürkle M., Zotti L. A., Asai Y., Pauly F., Cuevas J. C., Agraït N., Martín N. (2015). Beilstein J. Org. Chem..

[cit19] Kasibhatla B. S. T., Labonté A. P., Zahid F., Reifenberger R. G., Datta S., Kubiak C. P. (2003). J. Phys. Chem. B.

[cit20] Del Re J., Moore M. H., Ratna B. R., Blum A. S. (2013). Phys. Chem. Chem. Phys..

[cit21] Zhang W., Gan S., Vezzoli A., Davidson R. J., Milan D. C., Luzyanin K. V., Higgins S. J., Nichols R. J., Beeby A., Low P. J., Li B., Niu L. (2016). ACS Nano.

[cit22] Vezzoli A., Grace I. M., Brooke C., Nichols R. J., Lambert C. J., Higgins S. J. (2017). J. Chem. Phys..

[cit23] Kiguchi M., Nakashima S., Tada T., Watanabe S., Tsuda S., Tsuji Y., Terao J. (2012). Small.

[cit24] Nishino T., Ito T., Umezawa Y. (2005). Proc. Natl. Acad. Sci. U. S. A..

[cit25] Martín S., Grace I., Bryce M. R., Wang C., Jitchati R., Batsanov A. S., Higgins S. J., Lambert C. J., Nichols R. J. (2010). J. Am. Chem. Soc..

[cit26] Wu S., González M. T., Huber R., Grunder S., Mayor M., Schönenberger C., Calame M. (2008). Nat. Nanotechnol..

[cit27] Schneebeli S. T., Kamenetska M., Cheng Z., Skouta R., Friesner R. A., Venkataraman L., Breslow R. (2011). J. Chem. Soc., Chem. Commun..

[cit28] Ghane T., Nozaki D., Dianat A., Vladyka A., Gutierrez R., Chinta J. P., Yitzchaik S., Calame M., Cuniberti G. (2015). J. Phys. Chem. C.

[cit29] Frisenda R., Janssen V. A. E. C., Grozema F. C., van der Zant H. S. J., Renaud N. (2016). Nat. Chem..

[cit30] Fujii S., Tada T., Komoto Y., Osuga T., Murase T., Fujita M., Kiguchi M. (2015). J. Am. Chem. Soc..

[cit31] Guo C., Wang K., Zerah-Harush E., Hamill J., Wang B., Dubi Y., Xu B. (2016). Nat. Chem..

[cit32] Nishino T., Hayashi N., Bui P. T. (2013). J. Am. Chem. Soc..

[cit33] Chang S., He J., Kibel A., Lee M., Sankey O., Zhang P., Lindsay S. (2009). Nat. Nanotechnol..

[cit34] Nishino T., Shiigi H., Kiguchi M., Nagaoka T. (2017). Chem. Commun..

[cit35] Sautet P., Joachim C. (1988). Chem. Phys. Lett..

[cit36] Lambert C. J. (2015). Chem. Soc. Rev..

[cit37] Yang G., Sangtarash S., Liu Z., Li X., Sadeghi H., Tan Z., Li R., Zheng J., Dong X., Liu J.-Y., Yang Y., Shi J., Xiao Z., Zhang G., Lambert C., Hong W., Zhang D. (2017). Chem. Sci..

[cit38] Gantenbein M., Wang L., Al-jobory A. A., Ismael A. K., Lambert C. J., Hong W., Bryce M. R. (2017). Sci. Rep..

[cit39] Arroyo C. R., Tarkuc S., Frisenda R., Seldenthuis J. S., Woerde C. H. M., Eelkema R., Grozema F. C., van der Zant H. S. J. (2013). Angew. Chem., Int. Ed..

[cit40] Solomon G. C., Andrews D. Q., Van Duyne R. P., Ratner M. A. (2009). ChemPhysChem.

[cit41] Valkenier H., Guédon C. M., Markussen T., Thygesen K. S., van der Molen S. J., Hummelen J. C. (2014). Phys. Chem. Chem. Phys..

[cit42] Solomon G. C., Andrews D. Q., Goldsmith R. H., Hansen T., Wasielewski M. R., Van Duyne R. P., Ratner M. A. (2008). J. Am. Chem. Soc..

[cit43] Garner M. H., Li H., Chen Y., Su T. A., Shangguan Z., Paley D. W., Liu T., Ng F., Li H., Xiao S., Nuckolls C., Venkataraman L., Solomon G. C. (2018). Nature.

[cit44] Miao R., Xu H., Skripnik M., Cui L., Wang K., Pedersen K. G. L., Leijnse M., Pauly F., Wärnmark K., Meyhofer E., Reddy P., Linke H. (2018). Nano Lett..

[cit45] Breit G., Wigner E. (1936). Phys. Rev..

[cit46] Lambert C. J., Thorpe M. F. (1983). Phys. Rev. B: Condens. Matter Mater. Phys..

[cit47] Azbel M. Ya., Soven P. (1983). Phys. Rev. B: Condens. Matter Mater. Phys..

[cit48] Lambert C. J. (1984). J. Phys. C: Solid State Phys..

[cit49] Gefen Y., Imry Y., Azbel M. Y. (1984). Phys. Rev. Lett..

[cit50] Büttiker M., Imry Y., Landauer R., Pinhas S. (1985). Phys. Rev. B: Condens. Matter Mater. Phys..

[cit51] Magoga M., Joachim C. (1999). Phys. Rev. B: Condens. Matter Mater. Phys..

[cit52] Vazquez H., Skouta R., Schneebeli S., Kamenetska M., Breslow R., Venkataraman L., Hybertsen M. S. (2012). Nat. Nanotechnol..

[cit53] Borges A., Xia J., Liu S. H., Venkataraman L., Solomon G. C. (2017). Nano Lett..

[cit54] Ferraris J., Cowan D. O., Walatka V., Perlstein J. H. (1973). J. Am. Chem. Soc..

[cit55] Jerome D., Mazaud A., Ribault M., Bechgaard K. (1980). J. Phys., Lett..

[cit56] Frey J. E., Aiello T., Beaman D. N., Hutson H., Lang S. R., Puckett J. J. (1995). J. Org. Chem..

[cit57] Yoshida Z., Kobayashi T. (1970). Tetrahedron.

[cit58] Abu-Eittah R., Al-Sugeir F. (1976). Can. J. Chem..

[cit59] Xu B., Tao N. (2003). Science.

[cit60] Capozzi B., Dell E. J., Berkelbach T. C., Reichman D. R., Venkataraman L., Campos L. M. (2014). J. Am. Chem. Soc..

[cit61] Atesci H., Kaliginedi V., Celis Gil J. A., Ozawa H., Thijssen J. M., Broekmann P., Haga M., van der Molen S. J. (2018). Nat. Nanotechnol..

[cit62] Xu B., Fichou D., Horowitz G., Garnier F. (1991). Synth. Met..

[cit63] Hotta S., Kobayashi H. (1994). Synth. Met..

[cit64] Papadopoulos T., Grace I., Lambert C. (2006). Phys. Rev. B: Condens. Matter Mater. Phys..

[cit65] Soler J. M., Artacho E., Gale J. D., García A., Junquera J., Ordejón P., Sánchez-Portal D. (2002). J. Phys.: Condens. Matter.

[cit66] Perdew J. P., Burke K., Ernzerhof M. (1996). Phys. Rev. Lett..

[cit67] Ferrer J., Lambert C. J., García-Suárez V. M., Manrique D. Z., Visontai D., Oroszlany L., Rodríguez-Ferradás R., Grace I., Bailey S. W. D., Gillemot K., Sadeghi H., Algharagholy L. A. (2014). New J. Phys..

