# Supplementary material for: Charge transfer complexation boosts molecular conductance through Fermi level pinning
Source: Chem Sci. 2019 Jan 3;10(8):2396–403. doi: 10.1039/c8sc04199g (PMC6385675; doi:10.1039/c8sc04199g)
Supplement: Supplementary file 1 [file SC-010-C8SC04199G-s001.pdf]

Electronic Supplementary Material (ESI) for Chemical Science.
This journal is © The Royal Society of Chemistry 2019

ELECTRONIC SUPPLEMENTARY INFORMATION

Charge transfer complexation boosts molecular conductance through Fermi level pinning.

Kun Wang[a,≠], Andrea Vezzoli[b,≠], Iain M. Grace[c,≠], Maeve McLaughlin[b], Richard J. Nichols[b], Bingqian Xu[a,d*], Colin J. Lambert[c*] and Simon J. Higgins[b*]

a) Department of Physics and Astronomy & NanoSEC, University of Georgia, 220 Riverbend Road, Athens, GA 30602, U.S.A.

b) Department of Chemistry, University of Liverpool, Crown Street, Liverpool L69 7ZD, U.K.

c) Department of Physics, Lancaster University, Lancaster LA1 4YB, U. K.

d) College of Engineering & NanoSEC, University of Georgia, 220 Riverbend Road, Athens, GA 30602, U.S.A.

≠ these authors contributed equally to this work.

* corresponding authors: shiggins@liverpool.ac.uk, c.lambert@lancaster.ac.uk, bxu@engr.uga.edu.

**1. Synthetic procedures**	**S2**
**2. Additional Single-Molecule conductance data**	**S7**
**3. Additional theoretical calculation analysis**	**S12**
**4. References**	**S22**

1. Synthetic procedures

All reactions were performed in dry solvents (Thermofisher Scientific), in oven dried glassware, under Ar atmosphere, Solvents were dried and stored under activated 3 Å molecular sieves (20% m/v)[1] and DMF was degassed by three freeze-pump-thaw cycles. All reagents (Sigma-Aldrich Chemical Company, except where otherwise stated) were used without further purification except N-bromosuccinimide, which was recrystallized from boiling water. Alkyllithium compounds were titrated against benzylbenzamide to a blue endpoint before use.

Characterisation:

- Proton and ^{13}C NMR spectra were recorded using a Bruker Avance 400 Ultrashield spectrometer and referenced to internal TMS and the residual solvent peak. Coupling constants are given in Hz.
- High Resolution Mass Spectra (HRMS) was performed by the Liverpool University Analytical Services, using an Agilent QTOF 7200 spectrometer.
- CHNS microanalysis was performed by the Liverpool University Analytical Services, using an Elementar Vario Micro Cube spectrometer. Sulfur figure is inaccurate due to lack of an analytical standard for percentages > 18.57 % in the instrument employed.

Synthesis of precursors:

- 2,2':5',2''-Terthiophene was synthesised following published procedures[2] by Kumada coupling of freshly prepared 2-thienylmagnesium bromide and 2,5-dibromothiophene, using $NiCl_2$(dppe) as catalyst.
- 5,5'-Dibromo-2,2'-bithiophene was prepared from commercially available 2,2'-bithiophene (Manchester Organics), following the procedure developed by Bäuerle *et al.*[3]

- Trimethyl(5-(methylthio)thiophen-2-yl)stannane was prepared from thiophene (Acros Organics), by subsequent one-pot treatment with *n*-butyllithium and dimethyl disulfide, followed by *n*-butyllithium and trimethyltin chloride, adapting the procedure outlined by Barbarella *et al.*[4]

Preparation of T3-SAc:

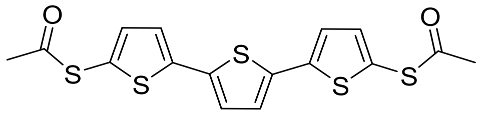

A solution of 2,2':5',2''-terthiophene (0.5 g, 2.01 mmol) in dry THF (40 mL) was cooled to -78 ºC and *n*-butyllithium (caution! 1.55 M, 4.00 mmol) was added dropwise whilst stirring. The yellow suspension was stirred for 30 minutes at that temperature and a suspension of S_8 (129 mg, 0.5 mmol) in THF (10 mL) was added dropwise. After further 60 minutes of stirring at -78 ºC, acetyl chloride (0.3 mL, 4.1 mmol) was added dropwise, and the mixture was allowed to return to room temperature and stirred overnight. Water (75 mL) and CH_2Cl_2 (15 mL) were then added, the phases were separated and the aqueous phase extracted with CH_2Cl_2 (3 x 30 mL). The combined organic phases were then washed with brine, dried over $MgSO_4$ and concentrated under vacuum. The resulting yellow crude product was then purified by column chromatography (10 % hexanes in CH_2Cl_2) to afford the title compound as bright yellow solid (0.577 g, 72 %). $C_{16}H_{12}O_2S_5$ requires C = 48.46, H = 3.05, S = 40.42 %. Found: C = 48.25, H = 3.06, S = 40.25 %. ^{1}H NMR (400 MHz, $CDCl_3$): 7.10 (s, 2H), 7.11 (d, J = 3.8 Hz, 2H), 7.07 (d J = 3.8 Hz, 2H), 2.43 (s, 6H) ppm. ^{13}C NMR (100 MHz, $CDCl_3$): 194.1, 142.9, 136.5, 136.1, 125.2, 124.3, 124.1, 29.6. *m/z* (HRMS, ES+, CH_3OH + NaOAc) 418.9347 $[M + Na]^+$, $C_{16}H_{12}NaO_2S_5$ calc. 418.9339.

Preparation of T2:

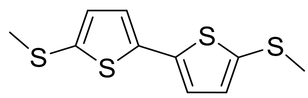

A solution of 2,2'-bithiophene (0.5 g, 3.01 mmol) in dry THF (40 mL) was cooled to -78 ºC and *n*-butyllithium (caution! 1.5 M, 6.00 mmol) was added dropwise whilst stirring. The dark green suspension was stirred for 30 minutes at that temperature and a suspension of S_8 (0.193 g, 0.75 mmol) in dry THF (10 mL) was added dropwise, and the mixture was allowed to return to room temperature and stirred for 60 minutes. The resulting red suspension was cooled down to -78 ºC before the addition of iodomethane (0.37 mL, 5.9 mmol) and then allowed to return to room temperature and stirred overnight. The resulting clear, honey coloured solution was quenched with water (75 mL), and extracted with CH_2Cl_2 (3 x 30 mL). The combined organic phases were dried over $MgSO_4$, filtered, and concentrated to dryness *in vacuo*. The crude yellow product was purified by column chromatography (10 % CH_2Cl_2 in hexanes) to give the title compound as a pale yellow solid (0.249 g, 32 %). Found: C = 46.46, H = 3.90 %. $C_{10}H_{10}S_4$ requires C = 46.47, H = 3.91 %. ^{1}H NMR (400 MHz, $CDCl_3$): 6.96 (m, 4H), 2.51 (s, 6H). ^{13}C NMR (100 MHz, $CDCl_3$): 139.2, 136.7, 131.6, 123.8, 22.1. *m/z* (HRMS, CI, CH_4) 258.9745 $[M + H]^+$, $C_{10}H_{11}S_4$ calc. 258.7944.

Preparation of T3:

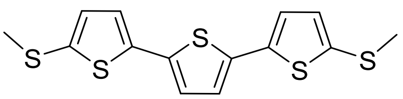

A solution of 2,2':5',2''-terthiophene (0.5 g, 2.01 mmol) in dry THF (40 mL) was cooled to -78 ºC and *n*-butyllithium (caution! 1.55 M, 4.00 mmol) was added dropwise whilst stirring. The yellow suspension was stirred for 30 minutes at that temperature and dimethyl disulfide (0.36 mL, 4.05 mmol) was added dropwise. The mixture was allowed to return to room temperature and was then stirred for 4 hours. Water (100 mL) and CH_2Cl_2 (30 mL) were added, the phases were separated,

and the aqueous phase was extracted with CH_2Cl_2 (3 x 25 mL). The combined organic phases were then washed with brine, dried over $MgSO_4$ and concentrated under vacuum. The resulting yellow crude product was then purified by column chromatography (20 % CH_2Cl_2 in hexanes) to afford a slightly impure (NMR) yellow solid. Recrystallisation from hot hexanes afforded the title compound as a bright yellow solid (0.329 g, 48 %). Found: C = 49.34, H = 3.53, S = 48.71 %. $C_{14}H_{12}S_5$ requires C = 49.37, H = 3.55, S = 47.08 %. ^{1}H NMR (400 MHz, $CDCl_3$): 7.01 (s, 2H), 7.00 (d, *J* = 3.8 Hz, 2H), 6.97 (d, *J* = 3.8 Hz, 2H), 2.51 (s, 6H) ppm. ^{13}C NMR (100 MHz, $CDCl_3$): 139.0, 136.6, 135.9, 131.8, 124.3, 123.7, 22.1 ppm. *m/z* (HRMS, CI, CH_4) 340.9632 $[M + H]^+$, $C_{14}H_{13}S_5$ calc. 340.9621.

Preparation of T4:

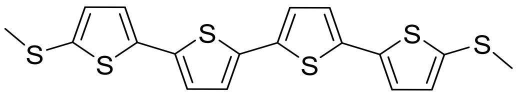

5,5'-Dibromo-2,2'-bithiophene (0.842 g, 1.49 mmol) was added to degassed DMF (30 mL), and the solution was sparged with Ar for 10 minutes. $Pd(PPh_3)_4$ (0.171 g, 0.149 mmol) was then added, and the solution sparged for additional 5 minutes. The reaction mixture was then heated to 85 ºC, and trimethyl(5-(methylthio)thiophen-2-yl)stannane (0.871 g, 2.98 mmol) was added. After stirring at 85 ºC overnight, dark orange crystals had crashed out of solution. The reaction mixture was then allowed to cool down to RT, the precipitate was collected by vacuum filtration and washed with $CHCl_3$ and methanol. ^{1}H NMR revealed the presence of a small amount of 5-bromo-5''-(methylthio)-2,2':5',2''-terthiophene contaminating pure **T4**, so the crude product was recrystallised from boiling DMF to afford the title compound in high purity as bright orange solid (0.246 g, 39%). Found: C = 51.11, H = 3.33 S = 42.33 %. $C_{18}H_{14}S_6$ requires C = 51.15, H = 3.34, S = 45.51 %. ^{1}H NMR (400 MHz, CDCl3): 7.06 (d, J = 3.9 Hz, 2H), 7.03 (d, J = 3.9 Hz, 2H), 7.01 (d, J = 3.4 Hz, 2H), 6.98 (d, J = 3.4 Hz, 2H), 2.52 (s, 6H). (HRMS, CI, CH_4) 422.9507 $[M + H]^+$,

$C_{18}H_{15}S_6$ calc. 422.9498. ^{13}C NMR spectrum was not obtained due to the low solubility of the compound, which resulted in poorly resolved spectra even with long acquisition times.

Further details on the synthesis of T4:

During the preparation of **T4**, several attempts were made at synthesising the compound by double lithiation of 5′,2′′:5′′,2′′′-quaterthiophene or halogen-lithium exchange on 5,5'''-dibromo-2,2':5',2'':5'',2'''-quaterthiophene and subsequent quench with either S_8/MeI or dimethyl disulfide, as in the procedures that successfully permitted the preparation of **T2** and **T3**. Unfortunately, in all these attempts, only the monosubstituted 5-(methylthio)-2,2':5',2'':5'',2'''-quaterthiophene was recovered, and the use of TMEDA or HMPA as additive failed to improve the yield. Similarly, treatment of 5,5'''-diiodo-2,2':5',2'':5'',2'''-quaterthiophene or 5,5'''-dibromo-2,2':5',2'':5'',2'''-quaterthiophene with sodium thiomethoxide in the presence of Cu(I) or Pd(0) catalysts resulted in the preparation of small amounts of the monosubstituted compound. Attempts at obtaining **T4** through homocoupling of 5-iodo-5'-(methylthio)-2,2'-bithiophene or 5-bromo-5'-(methylthio)-2,2'-bithiophene,[5] either through Kumada or Ullmann reaction, resulted in poor yields and unsatisfactory purity, well below the requirements for *STM-BJ* experiments. Suzuki coupling of (5'-(methylthio)-[2,2'-bithiophen]-5-yl)boronic acid and 5,5’-dibromo-2,2’-bithiophene was in fact successful, but the yield determined by crude NMR was deemed too low to make purification worthwile. All these issues can be attributed to the low solubility of substituted quaterthiophenes. Only a Stille coupling permitted the preparation of **T4**, which precipitates immediately upon formation and can be obtained in high purity and satisfactory yield.

2. Additional single-molecule conductance data

2.1 Effect of the electrode contact moiety:

To assess the effect of the terminal group used to contact the metallic electrodes, we synthesised and measured an α-terthienyl compound bearing thioacetate termini (**T3-SAc**), which cleave spontaneously in the presence of Au to give thiolate contacts.[6] The preparation of **T3-SAc** is described earlier, in section 1 of the SI.

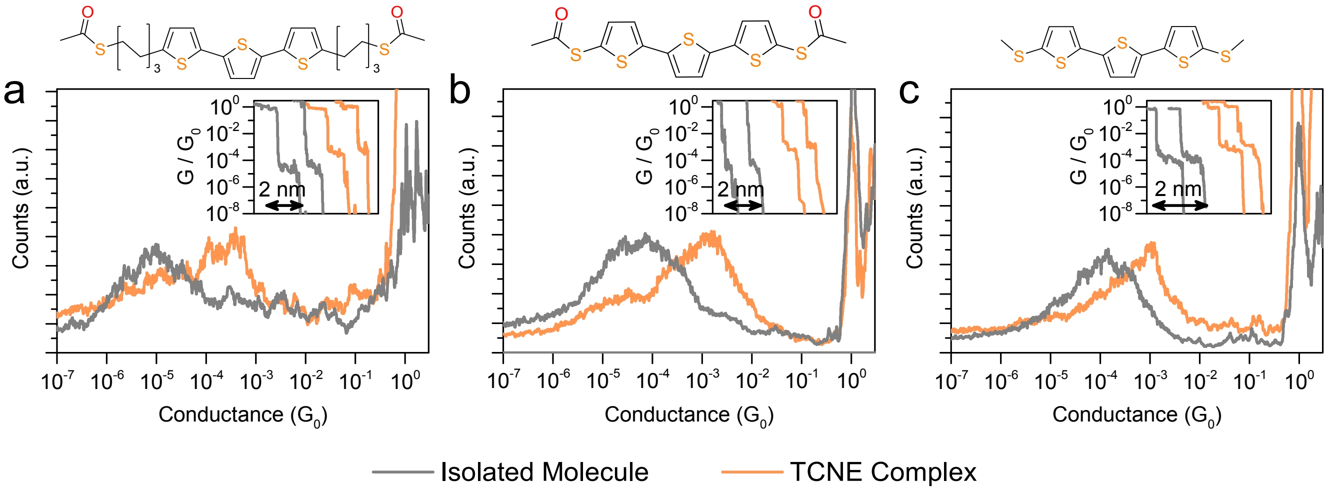

Figure S1: Effect of the contact to the electrodes on the conductance of α-terthienyl:TCNE complexes. Experimental conductance histograms for **6[T3]6**[7] (a), **T3-SAc** (b), and **T3** (c). Example conductance *vs.* electrode displacement traces are presented as inset. Data pertaining the isolated molecule is presented in grey, while the relative complex with TCNE is reproduced in orange.

A comparison of the previously characterised **6[T3]6**,[7] along with **T3-SAc** and **T3** are presented in **Figure S1**. While the three molecular wires have different conductance values when in their uncomplexed state (spanning the range 10^{-5} to $10^{-3.7}$ G_0), they all give very similar conductance values (~10^{-3} G_0) when TCNE is present. The least conductive α-terthienyl molecular wire **6[T3]6** showed the largest conductance boost upon complexation (~40-fold), while the most conductive compound **T3** had the smallest increase (10-fold). This is further proof that the charge transport

through the TCNE complexes is dominated by the quantum interference feature introduced by TCNE complexation, and that its energetic position, arising from a semi-occupied orbital, is pinned to E_F of the Au electrodes.

While for the thioether terminated **T3** there is good agreement between experiments and theory (see main manuscript), some discrepancies arise in the compounds terminated with thioacetate linker group, and good agreement can be obtained only at energies away from the electrode E_F (see **Section 3** of this document and our previous manuscript[7]). We attribute this issue to the thioacetate contact, which forms a charged thiolate on binding to gold and leads to a pinning of the HOMO to the Fermi energy, in competition with the CT Fano resonance. The DFT-predicted energy positions are therefore inaccurate, and some adjustment of the value of E_F is necessary to obtain good agreement with the experimental results. By using methyl thioether contacts, the molecule remains relatively unchanged upon interaction with the metallic lead and the Fermi energy is predicted to lie in the HOMO-LUMO gap. While confirming the proposed mechanism of conductance increase upon CT complexation, this also suggests that for a better agreement between theory and experiment, molecules with methyl thioether anchor groups are preferable.

2.2 Junction break-off statistical analysis

In the main text, we presented our data compiled in one-dimensional conductance histograms, which only represent the distribution of conductance values. Analysing the break-off length of the molecular plateau reveals no obvious variation upon CT complexation with TCNE. The average plateau lengths of **T2**, **T3** and **T4** are around 0.9, 1.3 and 1.7 nm, which correlates well with the molecular S-S distances calculated by DFT, which are, respectively, 1.004, 1.398 and 1.795 nm. The average plateau length of **T3-SAc** and **6[T3]6** are around 1 and 2.2 nm, respectively.

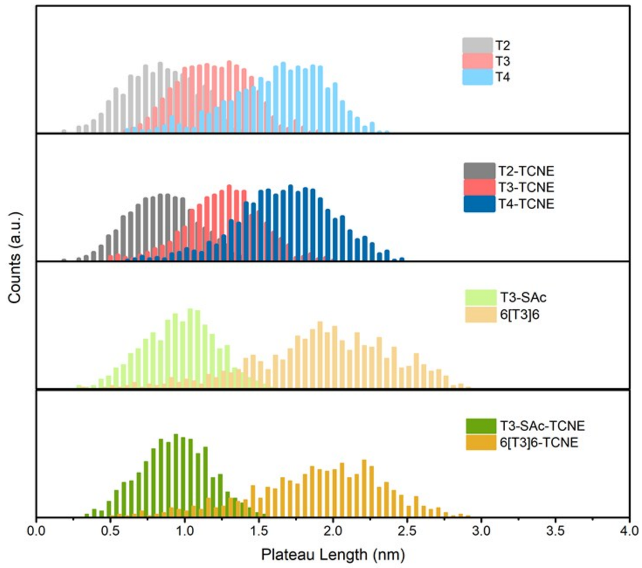

Figure S2: Single-molecule plateau length analysis for all molecular compounds studied in the main text.

2.3 Control experiment: STM-BJ of clean Au and TCNE adsorbed on Au.

As discussed in the main paper, we performed a control experiment to rule out the participation of TCNE alone (and not its CT complexes with the oligothiophene molecular wire) could be responsible for the increase in conductance. Results are presented in **Figure S3**.

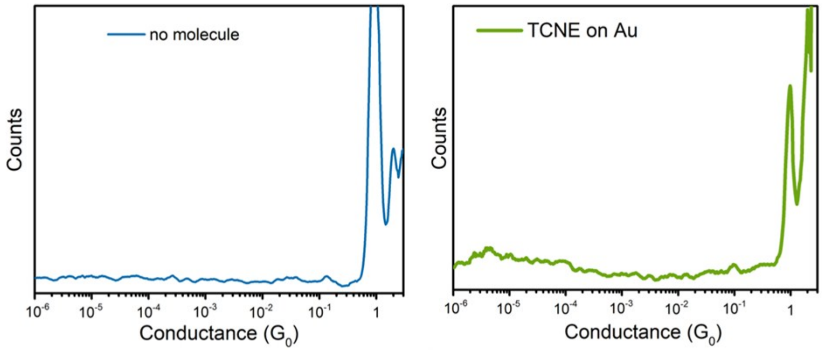

Figure S3. Control experiments performed on sample with no molecule on Au surface (left: blue histogram) and sample solely with TCNE on Au surface (right: green histogram).

We note that there is no significant feature observed within the range of 10^{-5} to 1 G_0, which rules out possible contribution from interactions of TCNE with the Au electrodes to the higher conductance peak at around 10^{-3} G_0 observed in Figure 2 and 3 of the main text.

2.4 Effect of TCNE adsorption and changes in the workfunction on conductance.

The effect of TCNE on the conductance of molecular wires not able to form CT complexes was examined previously.[7] In brief, we measured the conductance of 1,10-decanedithiol in the presence of excess TCNE, and we found no significant increase in conductance. This experiment showed that any change in workfunction due to TCNE adsorption[8] at the two electrodes in our experiments results in negligible conductance changes, and therefore it is indeed an interaction between TCNE and the molecular wire π-system promoting the observed conductance increase. Furthermore, changes in the workfunction of the electrodes due to solvent resulted in only a factor of < 2-fold conductance changes even with a short, π-conjugated molecule (benzene-1,4-diamine),[8] while in our case we observed up to 70-fold increase when TCNE is complexed with the molecular wire. Such a large value is not consistent with a simple change in workfunction, especially for the longest molecular wires. Taking T4 as an example, its G(E) curve is rather flat in the region around E_F (Figure 4c), and even a change of 0.5 eV in the workfunction would not explain the exceptional conductance increase we observed.

3. Additional theoretical calculations details

An exemplary model Hamiltonian is presented in Papadopoulos *et al.*[9] which describes a pendant orbital of energy ϵ, coupled to a backbone orbital of energy ϵ_0 by a coupling matrix element ω. The transmission coefficient through such a molecule is predicted to be

$$T(E) = \frac{4\Gamma_1\Gamma_2}{[(E - \epsilon_1)^2 + (\Gamma_1 + \Gamma_2)^2]}$$ (equation S1)

where $\epsilon_1 = \epsilon_0 + \frac{\omega^2}{E - \epsilon}$ and Γ_1, Γ_2 are the level broadenings due to the contacts between the backbone orbital and the electrodes. Clearly a resonance occurs when $E = \epsilon_1$ and a nearby anti-resonance occurs when $E = \epsilon$, (ie when $\epsilon_1 = \infty$ and therefore $T(E) = 0$), which combine to yield an asymmetric lineshape.

3.1 Molecular orbitals calculated using DFT

	T3SAc	T2	T3	T4
	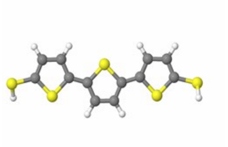	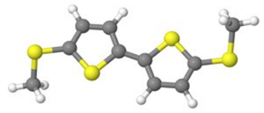	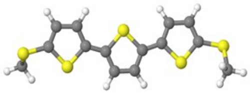	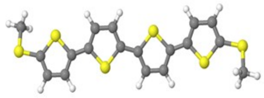
HOMO-1	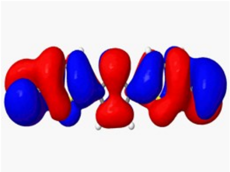 -4.86eV	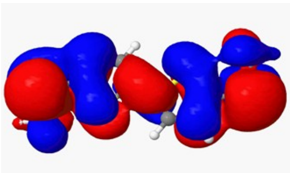 -4.80eV	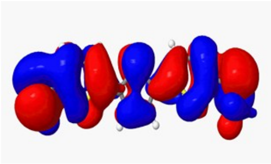 -4.63eV	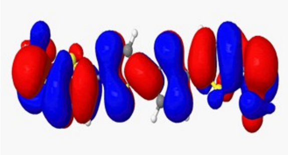 -4.33eV
HOMO	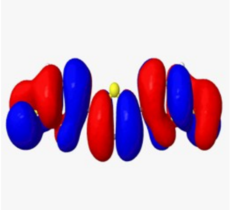 -3.98eV	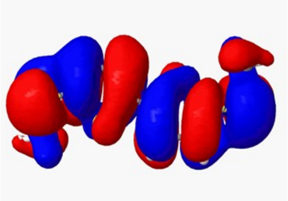 -3.83eV	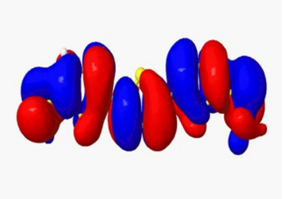 -3.88eV	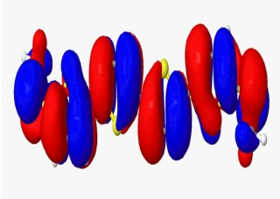 -3.67eV
LUMO	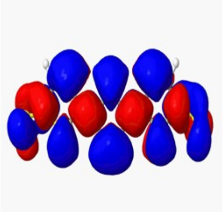 -1.92eV	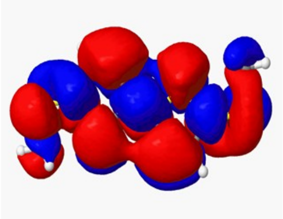 -1.38eV	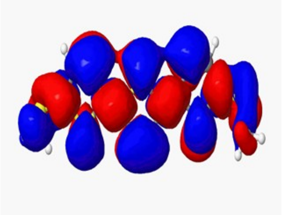 -1.83eV	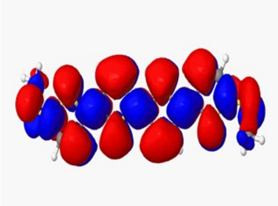 -1.94eV
LUMO+1	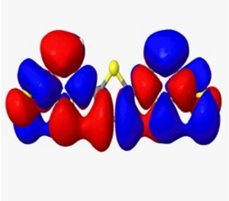 -0.77eV	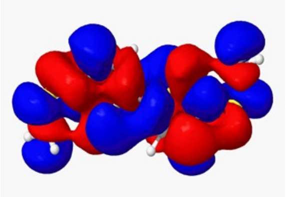 0.20eV	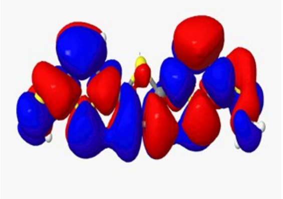 -0.69eV	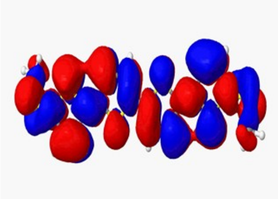 -1.06eV

3.2 Ionization Potential and Electron Affinity Calculations

The Ionization Potential (IP) and electron affinity (EA) of the series of molecules is calculated with SIESTA using the following formula: IP = E(N-1) – E(N) and EA = E(N) – E(N+1), where E is the ground state energy of the molecule and N is the number of electrons. In this case the values are calculated in the gas phase with the **T3-SAc** anchor groups deprotected but maintaining their hydrogen atom (as thiols), which is not the case when this molecule is contacted to the gold electrodes. The results are shown in **Table S1**.

	**IP (eV)**	**EA (eV)**
**T2**	5.98	-0.87
**T3**	5.74	-0.09
**T4**	5.32	0.236
**T3-SAc**	6.093	0.02
**T2:TCNE**	6.21	1.64
**T3:TCNE**	5.88	1.64
**T4:TCNE**	5.51	1.67
**T3-SAc:TCNE**	5.92	1.62

Table S1: DFT calculated Ionization Potentials and electron affinities of **T2-T4**, **T3-SAc** and their charge-transfer complexes with TCNE.

As can be inferred from the table above, the ionisation potentials of the **T2-T4** series are all raised by 0.2 - 0.3 eV when complexed with **TCNE.** This is consistent with the notion that there is a partial molecule→TCNE electron transfer, as the CT complex is generated, and that the electron can only be removed from the electron-rich oligothiophene (or, rather, only the oligothiophene orbitals are affected by the removal of an electron). This is demonstrated in the table below, which

shows that the Mulliken population of the TCNE is approximately +1, when an electron is added to the complex (*i.e.* N = 1). On the other hand, the Mulliken population on the TCNE barely changes when an electron is removed. Consequently, the calculated ionisation potential is variable.

We calculate the Mulliken charge on the TCNE molecule when it forms the charge transfer complex. This is done for three cases, when the number of added electrons to the complex is N = 0, N = -1 and N = 1, to explain the behaviour of the electron affinity and ionization potential when the complex is formed. The TCNE molecule in isolation contains 44 valence electrons.

	**N = 0**	**N = -1**	**N = 1**
**T2:TCNE**	44.14	44.0	44.97
**T3:TCNE**	44.16	44.02	44.9
**T4:TCNE**	44.22	44.03	44.8
**T3-SAc:TCNE**	44.19	44.02	44.9

Table S2: Change in the number of electrons on the TCNE molecule when it forms a charge transfer complex. For the neutral case (N = 0), when an electron is removed (N = -1) and an electron is added (N = 1)

3.3 Geometry of charge transfer complexes

The optimum geometry of the charge transfer complexes with TCNE are calculated by minimising the geometry with respect to the binding energy using the method described earlier. The separation between the TCNE and thiophene is calculated to be 0.31 nm for **T2**, 0.32 nm for **T3**, 0.31 nm for **T3-SAc** and 0.33 nm for **T4**.

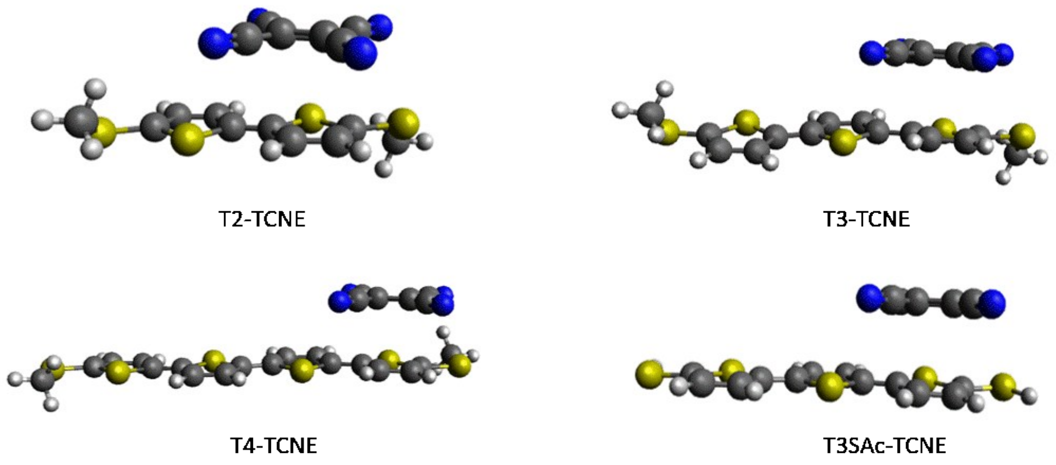

Figure S4: Optimum binding locations for the TCNE molecule when the charge transfer complex is formed with the T2, T3, T4 and T3SAc molecules.

3.4 Binding Energy Calculations

To calculate the binding energy between the TCNE molecule and the thiophene based wires we use the counterpoise method, which removes basis set superposition errors (BSSE) found in using a finite size basis DFT code. Here, the TCNE is defined as entity A and the molecule as entity B. The ground state energy of the total system was calculated using SIESTA and is denoted E^{AB}_{AB}, with the DFT parameters defined previously. The energy of each entity was then calculated in a fixed basis, which was achieved through the use of ghost atoms in SIESTA. Hence, the energy of the individual TCNE molecule in the presence of the fixed basis is defined as E^{AB}_{A} and for the molecule as E^{AB}_{B}. The binding energy was then calculated using the following equation:

$$BindingEnergy = E^{AB}_{AB} - E^{AB}_{A} - E^{AB}_{B} \quad \text{(equation S2)}$$

The resulting binding energy for the geometry of the optimum complexes are as follows:

T2:TCNE = 0.528 eV

T3:TCNE = 0.507 eV

T4:TCNE = 0.545 eV

T3-SAc:TCNE = 0.475 eV.

3.5 Zero bias transmission calculations

Below (**Figure S5**) are the zero temperature, spin polarized calculations of T(E) for the optimum geometries shown in the calculated transmission for the up spin (T_up) and down spin (T_down) show that the Fano resonances are split as expected due to the charge transfer from the thiophene to the TCNE which positions the Fano resonance close to the Fermi energy. The total transmission is then given by $\frac{T_{up} + T\_down}{2}$.

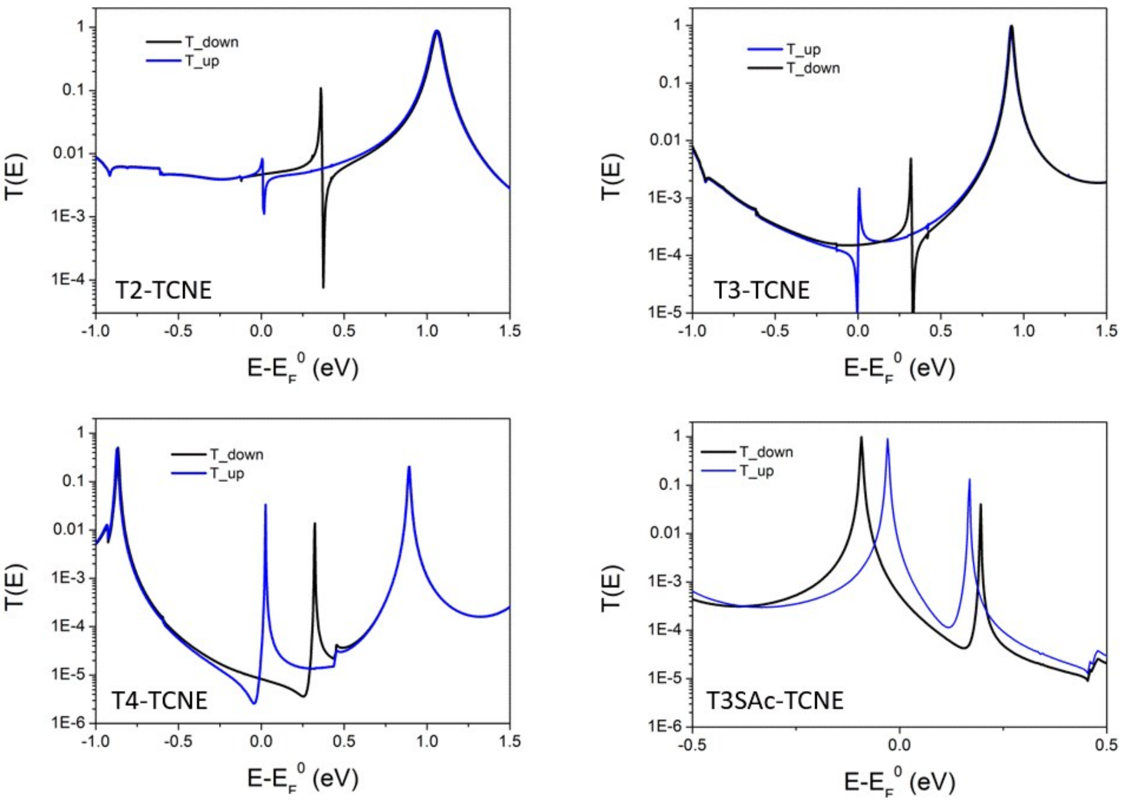

Figure S5: Zero bias transmission coefficients for the up and down spins for the optimum charge transfer complexes shown in **Figure S4**.

3.5 Molecular dynamic simulations of charge transfer complexes

The MD simulations are performed using SIESTA at a constant temperature and volume (NVT). We equilibrate the junction for 1 ps with 1 fs time steps, for a temperature of 290 K and then allow the system to run for another 2 ps. The calculations are performed with the same parameters as for the geometry optimizations. The initial geometry is taken to be the optimum geometry from DFT including the pyramid of gold atoms, which are constrained to not move while the complexed molecule is free to. We then use the MD coordinates to calculate the conductance, 500 snapshots of the junction at 5 fs intervals are taken and for each snapshot we feed the atomic coordinates into the DFT code SIESTA and generate the DFT Hamiltonian. The thermally averaged conductance is then computed in the same way as described in the main text.

Figure S6 Shows the comparison for this approach compared to the fixed geometry model used in the main paper in the case of charge transfer complex **T3:TCNE**. The overall trend is the same with the spin split Fano resonances forming two broadened peaks at the same energies close to the Fermi energy (0 eV). The difference in the magnitude, the MD calculation provides a higher conductance is due to the fact that the whole complex is free to move, so not only is the position of the TCNE molecule changing but also the geometry of the T3 and the binding geometry.

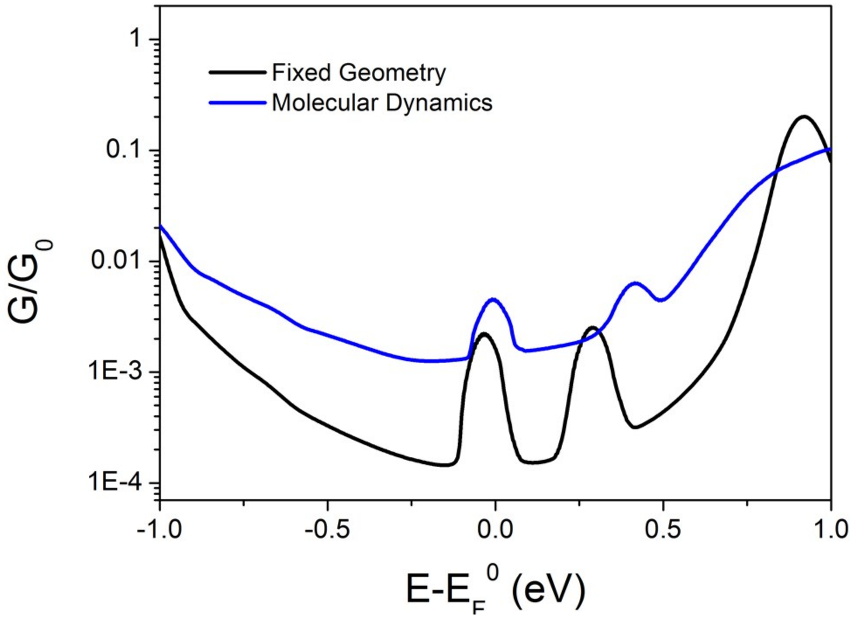

Figure S6: Room temperature conductance of the T3-TCNE complex using two different models. (Black line) A fixed geometry for the T3 molecule with the TCNE geometry moved through a range of binding locations and (blue line) a molecular dynamics simulation of the T3-TCNE molecule.

3.6 Local density of States (LDOS) for the T4:TCNE Complex

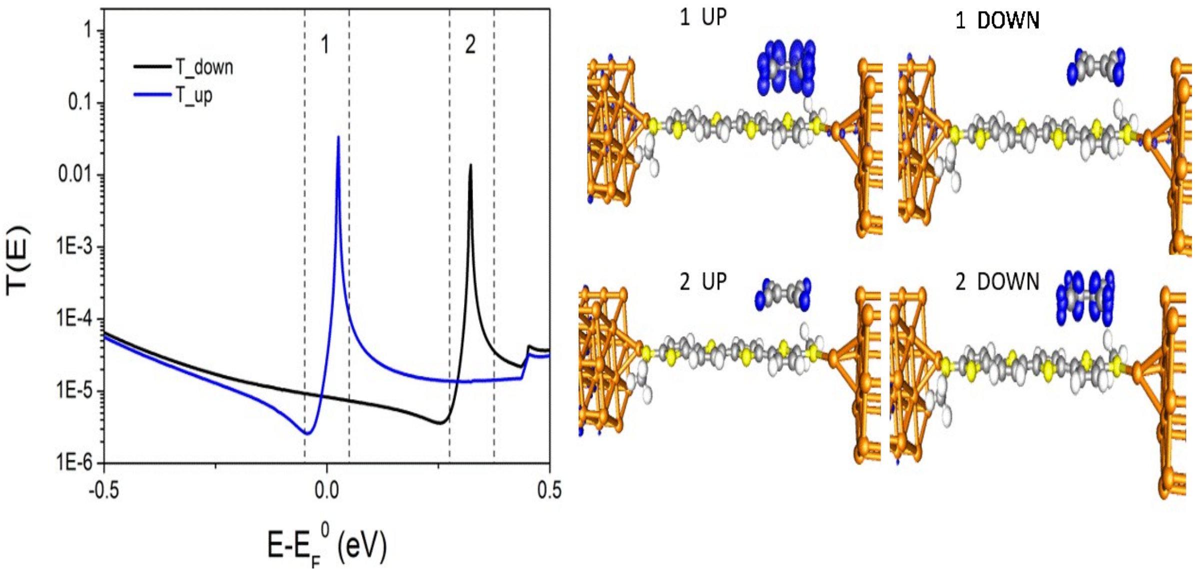

Figure S7: (Left) Zero bias transmission coefficient T(E) for compound **T4** complexed with TCNE for the up and down spins. (Right) Structure and LDOS evaluated for the energy windows 1 and 2 for the up and down spins.

The transmission coefficient T(E) for the complexed molecule shows a Fano shaped resonance at $E - E_F^0 = 0$ eV for the up spin and at $E - E_F^0 = 0.3$ eV for the down spin. We attribute this behaviour to the TCNE which due to charge transfer leads to a part-filled orbital which must be necessarily located near to the Fermi energy $E - E_F^0 = 0$ eV. To show this we evaluate the LDOS in two energy windows shown in **Figure S7** and plot contours of constant value in blue. Firstly, region **1** between -0.05 eV and 0.05 eV shows for the up spin the orbital is located on the TCNE molecule while for the down spin there is no weighting of the LDOS as it is off resonance. Secondly, in the region 0.275 to 0.375 eV the up spin shows no weight while the down spin shows the LDOS located on the TCNE.

4. References

1 D. B. G. Williams and M. Lawton, *J. Org. Chem.*, 2010, **75**, 8351–4.

2 B. J. J. Smeets, R. H. Meijer, J. Meuldijk, A. J. M. Vekemans and L. A. Hulshof, *Org. Process Res. Dev.*, 2003, **7**, 10–16.

3 P. Bauerle, F. Wuerthner, G. Goetz, F. Effenberger, P. Bäuerle, F. Würthner, G. Götz and F. Effenberger, *Synthesis (Stuttg).*, 1993, **1993**, 1099–1103.

4 G. Barbarella, M. Zambianchi, A. Ventola, E. Fabiano, F. Della Sala, G. Gigli, M. Anni, A. Bolognesi, L. Polito, M. Naldi and M. Capobianco, *Bioconjug. Chem.*, 2006, **17**, 58–67.

5 M. Tateno, M. Takase and T. Nishinaga, *Chem. Mater.*, 2014, **26**, 3804–3810.

6 A. Singh, D. H. Dahanayaka, A. Biswas, L. A. Bumm and R. L. Halterman, *Langmuir*, 2010, **26**, 13221–13226.

7 A. Vezzoli, I. Grace, C. Brooke, K. Wang, C. J. Lambert, B. Xu, R. J. Nichols and S. J. Higgins, *Nanoscale*, 2015, **7**, 18949–18955.

8 V. Fatemi, M. Kamenetska, J. B. Neaton and L. Venkataraman, *Nano Lett.*, 2011, **11**, 1988–92.

9 T. Papadopoulos, I. Grace and C. Lambert, *Phys. Rev. B*, 2006, **74**, 193306.
